# A Novel Ca-Modified Biochar for Efficient Recovery of Phosphorus from Aqueous Solution and Its Application as a Phosphorus Biofertilizer

**DOI:** 10.3390/nano12162755

**Published:** 2022-08-11

**Authors:** Yue Xu, Huan Liao, Jing Zhang, Haijun Lu, Xinghua He, Yi Zhang, Zhenbin Wu, Hongyu Wang, Minghua Lu

**Affiliations:** 1 School of Civil Engineering and Architecture, Wuhan Polytechnic University, Wuhan, 430023, China; 2 State Key Laboratory of Freshwater Ecology and Biotechnology, Institute of Hydrobiology, Chinese Academy of Sciences, Wuhan 430072, China; 3 School of Civil Engineering, Wuhan University, Wuhan 430072, China; 4 College of Chemistry and Chemical Engineering, Henan University, Kaifeng 475004, China

**Keywords:** phosphorous, oyster shell, peanut shell, biochar, P biofertilizer

## Abstract

Recovery phosphorus (P) from P-contaminated wastewater is an efficient and environmentally friendly mean to prevent water pollution and alleviate the P shortage crisis. In this study, oyster shell as calcium sources and peanut shells as carbon sources (mass ratio 1:1) were used to prepare a novel Ca-modified biochar (OBC) via co-pyrolysis, and its potential application after P adsorption as a P biofertilizer for soil was also investigated. The results shown that OBC had a remarkable P adsorption capacity from wastewater in a wide range of pH 4–12. The maximum P adsorption capacity of OBC was about 168.2 mg/g with adsorbent dosage 1 g/L, which was about 27.6 times that of the unmodified biochar. The adsorption isotherm and kinetic data were better described by Langmuir isotherm model (R^2^ > 0.986) and the pseudo second-order model (R^2^ > 0.975), respectively. Characterization analysis of OBC before and after P adsorption by scanning electron microscopy (SEM), X-ray diffraction (XRD), Fourier transform infrared spectroscopy (FTIR), and specific surface area and porosity analyzer (BET) indicated that the remarkable P adsorption capacity of OBC was mainly ascribed to chemical precipitation, electrostatic adsorption, and hydrogen bonding. Pot experiment results showed that OBC after P adsorption could significantly promote the germination and growth of *Spinacia*, which manifested that OBC after P adsorption exhibited a good ability to be reused as P fertilizer for soil.

## 1. Introduction

In recent years, with the rapid development of industrialization and domestic urbanization, excessive nitrogen (N) and phosphorus (P) are discharged into surface water by agricultural runoff and sewage effluent, which can lead to eutrophication, diminish the self-purification capacity of aquatic environment, destroy the ecological balance and harm human’s health [1,2,3,4,5,6,7,8]. By a 37-year experiment on nutrient management in Canadian lakes, Schindler [9] found that P input directly controlled algal reproduction and was a key factor in causing eutrophication. Moreover, many studies also found that most of eutrophic lakes were P-controlled, and very few lakes were N-controlled [10,11]. Hence, P control is of great significance to water pollution prevention.

According to “China’s Ecological and Environmental Status Bulletin (2019)”, the eutrophication rate of China’s lakes (reservoirs) was about 28% in 2019, and the main pollution index was total phosphorus (TP). In general, water bodies will undergo eutrophication if the concentration of total TP exceeds 0.02 mg/L [12]. Unfortunately, the P concentration of the discharges from wastewater treatment plant (WWTP) and agricultural runoff is often much higher than 0.02 mg/L [12,13]. Hence, an increasingly stringent P discharge criteria have been enacted around the world. For example, the new P discharge criteria for WWTP in Europe and in North America are 0.05 mg/L and 0.0 1mg/L, respectively. Moreover, as an essential nutrient in the growth and development of humans and other organisms, P is a non-renewable resource. It is reported that P rocks as the main source of element P would be exhausted in 300 years [12]. Therefore, the high-level P of wastewater also can be regarded as a source of P; and thus, an efficient recovery of P from wastewater not only can alleviate the P shortage crisis, but also can prevent eutrophication of water bodies.

Now many effective physical, chemical, and biological methods have been applied for P removal/recovery from wastewater [7,14,15,16,17]. Biological phosphorus removal method mainly depends on the metabolism of some microorganisms to remove P form wastewater. Due to high P removal efficiency, low cost and low sludge yield, biological phosphorus removal method is regarded as a promising method for the removal of P from wastewater. Nevertheless, biological phosphorus removal method is only suitable for wastewater with low P concentration, and its P removal rate is generally only 30% to 40%. Chemical phosphorus removal method achieves the purpose of P removal by precipitation reaction or ion exchange between the added chemical reagents and P in wastewater. It has an excellent P removal effect for wastewater even with high concentration of P. However, adding chemical reagents not only can increase its economic costs, but also can cause sludge contamination. Compared with the previous two methods, adsorption not only exhibits high efficiency, simplicity in operation and minimal sludge production, but also can be applied to wastewater treatment with various concentrations. So, adsorption has attracted more and more attention around the world [7,18]. Up to now, many adsorbents for adsorption, such as bentonite, zeolite, activated carbons, biochar, and so on, have been used for P removal, and shown different P removal efficiency [19,20,21,22,23,24]. Among them, because of high surface, well-developed pore and richness of surface functional groups, biochar has been seen as an economical, practical, and environmentally friendly adsorbent and soil conditioner [22,25]. However, the high hydration energy of the P anion, negative charge of biochar, and coexisting anions in wastewater pose a great obstacle for biochar to carry out the selective sorption of P from an aqueous phase [26,27]. Therefore, the development of biochar with high P selectivity is the key to achieving high level P removal/recovery.

According to the theory of hard soft acid base (HSAB) [28], P as a hard base could react fast with hard acid (as Fe^3+^, Al^3+^, Mg^2+^, Ca^2+^) and form a strong bond [26]. That is, metal-modified biochar can achieve selective P adsorption. It is reported that metal cations, such as Ca^2+^, Mg^2+^, Fe^3+^, and La^3+^, have strong affinity with P, which can significantly improve the P adsorption efficiency for biochar [27,29,30,31,32]. Among them, Ca is an ideal metal element for biochar modification because of its low cost, ecologically non-toxic property, and abundancy in nature [27,33]. Relevant studies have proved that the P adsorption capacity of Ca-modified biochar is remarkably improved [27,34]. However, if a large number of calcium reagents are used for biochar modification, it will lead to high cost and a large amount of calcium waste, so more economic and friendly environmental raw rich-Ca materials are needed [27]. Oyster is a kind of economic marine shellfish with huge yield, and its shell contains approximately 96% calcium carbonate, which is a kind of cheap and rich-Ca biological waste [35,36]. Therefore, oyster shell has great potential as a Ca source for the preparation of Ca-modified biochar.

In this research, the oyster shell waste was used as the Ca source, and the peanut shell was used as the C source to prepare Ca-modified biochar (OBC) for the removal/recovery of P from aqueous solution. This work was conducted by assessing the effect of initial pH on the adsorption of P by OBC. The kinetic and equilibrium behaviors during the P adsorption process were also investigated to determine the P adsorption performance and mechanism of OBC. Furthermore, the changes in surface chemical and space pore structures of OBC during P adsorption were characterized using XRD, FTIR, SEM, and BET. At last, the potential as a P fertilizer of OBC for soil after P adsorption was studied by a pot experiment. The experiment results indicated that the prepared Ca-modified biochar exhibited excellent P adsorption performance from aqueous solution and had a good ability as P fertilizer for soil after P adsorption. This work provided a promising method to prepare functionalized biochar from agricultural wastes, which has wide applications in P removal/recovery and agricultural application for P resource.

## 2. Materials and Methods

### 2.1. Materials

Peanut shell as biochar precursor was collected from a farm in Wuhan, Hubei Province, China. Oyster shell was obtained from an Oyster farm in Dandong, Liaoning Province, China. Both of them were washed, naturally dried, crushed, and sieved through a 100-mesh sieve. Then they were dried at 60 °C and sealed in a container before experiments. Potassium phosphate monobasic (KH_2_PO_4_) and other chemical reagents were of analytical grades and purchased from Shanghai Aladdin Biochemical Technology Co., Ltd. (Shanghai, China).

### 2.2. Preparation of Experiment Materials

Ca-biochar was prepared by co-pyrolysis of oyster shell and peanut shell in a programmable tube electric furnace (OTF-1200X, Jingke, Hefei, China) at 800 °C. Oyster shell and peanut shell powder were fully mixed with mass ratio of 1:1, then placed in the programmable tube electric furnace and heated up to 800 °C with heating rate of 10 °C/min and holding time of 2 h under N_2_ atmosphere. The synthesized material was denoted as OBC. Meanwhile, an unmodified biochar (BC) only with peanut shell powder was prepared under the same condition. Stock solution (1000 mg P/L) was prepared by dissolving KH_2_PO_4_ in deionized water, and the solutions for the following experiment were prepared by diluting the stock solution.

### 2.3. P Adsorption

The performances of OBC and BC as P sorbents in this study were investigated by batch experiments in duplicates. Adsorption isotherm of P for OBC was investigated by adding 0.02 g different sorbent into 20mL different initial P concentrations (0, 10, 25, 50, 100, 200, 300, 400 mg/L), and shaken at 180 rpm at the temperature of 28 ± 0.5 °C for 24 h in a mechanical shaker (QYC-200, Shanghai, China). Afterwards, the mixtures were filtered by a 0.45-μm PES syringe filter and the final P concentration after adsorption was determined by ammonium molybdate spectrophotometric method using an ultraviolet spectrophotometer at a wavelength of 700 nm (SP-1920, Shanghai, China) and by a pH Meter (BPH-7200, Dalian, China), resp. According to the P concentration difference in solution before and after adsorption experiments, the P adsorption capacity of the sorbents were calculated by the same mass balance equation used in Qu’s study [37].

Adsorption kinetics were addressed by mixing 0.02 g of different sorbent into 50 mL polyethylene tubes with 20 mL of 300 mg/L P solution. Then these containers were shaken using a shaker with the rate of 180 rpm at the temperature of 28 ± 0.5 °C for appropriate sampling time (e.g., 2, 6, 15, 30, 60, 180, 360, 720, and 1440 min). 

To investigate the effect of initial pH on P adsorption, 20 mL of 300 mg/L P solution with different initial pH (2, 4, 6, 8, 10, 12) was added into 50 mL polyethylene tube. The initial pH of the solution was adjusted by 0.1 M NaOH and 0.1M HNO_3_ solutions. Then, 0.02 g of adsorbent was added and shaken using a shaker with the rate of 180 rpm at a temperature of 28 ± 0.5 °C for 24 h; after that, the mixed solution was filtered through a 0.45 μm PES syringe filter, and the final P concentration and final pH in the filtrate were measured using an ultraviolet spectrophotometer at a wavelength of 700 nm (SP-1920, Shanghai, China) and a pH Meter (BPH-7200, Dalian, China), respectively. Then, the P adsorption capacity of the sorbents were calculated by the same mass balance equation used in Qu’s study [37].

### 2.4. Characterization

The micro-morphology, surface functional group, crystal structure, and pore structure of biochar samples were characterized by scanning electron microscopy (SEM, Mira Lms, Tescan, Brno, South Moravia, Czech Republic), Fourier transform infrared spectroscopy (FTIR, Nicolet 6700, Thermo Fisher Scientific, Waltham, MA, USA), X-ray diffraction (XRD, D8 advance, Bruker, Karlsruhe, Germany), and specific surface area and porosity analyzer (ASAP2020, Mike, Atlanta, GA, USA), respectively.

### 2.5. Seed Germination and Seedling Growth

A pot experiment was conducted to investigate the application of OBC after P adsorption as a P fertilizer for the plant. First, market-bought *Spinacia* seeds were washed with DI water three times. Second, soil collected from a farmland in Henan Province was dried in an oven at 60 °C for 24 h, and then screened with a 50-mesh sieve. The dried OBC after P adsorption from the adsorption isotherm experiment was manually mixed with 100 g dried soil in 3.0 × 2.3 × 7.5 cm plastic boxes. The mass ratio of OBC after P adsorption was 0.25% and 0.5%. Meanwhile, a group only with 100 g of dried soil was taken as the blank control. Ten treated *Spinacia* seeds were placed in each box. The seeds were completely covered in the medium. The *Spinacia* seeds in all the plastic boxes were cultivated under similar conditions such as natural light and the soil moisture was kept at constant levels by distilled water. The condition of germination and growth was recorded by taking photos every day, and the plants were collected for statistical analysis after 15 days.

## 3. Results and Discussion

### 3.1. P Adsorption Experiments

#### Effects of Initial P Concentration and Adsorption Isotherms

In this study, the well-known isotherm models Langmuir (Equation (1)), Freundlich (Equation (2)), and Temkin (Equation (3)) were used to investigate the P adsorption behavior on the prepared OBC samples [38].

(1) $$\mathrm{Langmuir}:\;Q_{e}=K_{L}\cdot q_{max}\cdot C_{e}/\left(1+K_{L}\cdot C_{e}\right)$$

(2) $$\mathrm{Freundlich}:\;Q_{e}=K_{F}C_{e}^{1/n}$$

(3) $$\mathrm{Temkin}:\;Q_{e}=\frac{RT}{b_{T}}\ln\left(K_{T}C_{e}\right)$$

where $Q_{e}$ (mg/g) is the equilibrium P adsorption capacity. $C_{e}$ (mg/L) is the equilibrium concentration of the sorbents. $q_{max}$ (mg/g) is the maximum adsorption capacity. $K_{L}$ and $q_{max}$ (mg/g) are the Langmuir equation constants relating to the sorption energy and maximum sorption capacity, respectively. $K_{F}$ and *n* are the Freundlich equation constants relating to the capacity and intensity of the sorption, respectively. T (K) is the absolute temperature, R = 8.314 J mol/K. $b_{T}$ and $K_{T}$ are adsorption heat and equilibrium binding constant, respectively.

**Figure 1:**
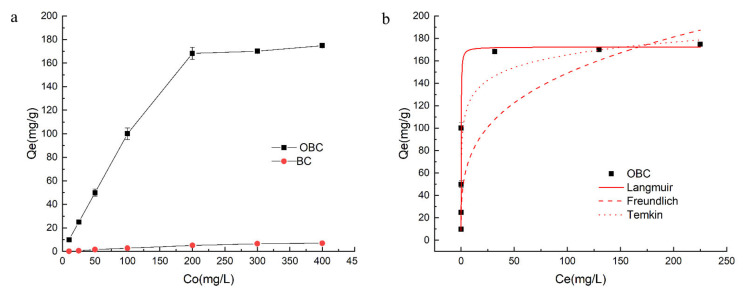
Adsorption capacity (**a**) and adsorption isotherms (**b**) of synthetic materials.

The effects of initial P concentration on P adsorption capacity and the three adsorption fitting curves of OBC are depicted in Figure 1a,b, respectively. Initial concentration of adsorbed substance and the number of surface-active sites on adsorbent were the two crucial factors for the adsorption. Initial concentration of adsorbed substance could provide an important driving force to overcome the mass transfer resistance between liquid phase and the solid adsorbent surface. The number of surface-active sites onto adsorbent can determine its the maximum adsorption capacity. In this experiment, the dosage of adsorbent was 0.02 g, thus the total number of surface-active sites onto adsorbent was definite. Moreover, the amount of P that can be absorbed was also definite. As shown in the Figure 1a, there were enough surface-active sites on OBC to adsorb all P in the solution with low initial P concentration, so the P equilibrium adsorption capacity increased with the increase in initial P concentration from 10 mg/L to 200 mg/L. Otherwise, when the surface-active sites on OBC were gradually occupied by P, even though more P were still in the water, there were no redundant surface-active sites to adsorb P. Thus, when the initial P concentration exceeded 200 mg/L, the adsorption capacity almost kept stable. Hence, the initial P concentration of 200 mg/L was a demarcation point for the P adsorption capacity of OBC. Moreover, the maximum P adsorption capacity of OBC was 168.2 mg/g, which was about 27.6-folds of BC. 

The fitting parameters of three different adsorption models of OBC are listed in Table 1. Evidently, the nonlinear correlation coefficients of the Langmuir, Freundlich, and Temkin model for OBC are 0.987, 0.960, and 0.982, respectively. The results indicated the entire P adsorption process by OBC was most consistent with the Langmuir model. This suggested the adsorption of P onto OBC belonged to single molecular layer adsorption [39]. 

**Figure 2:**
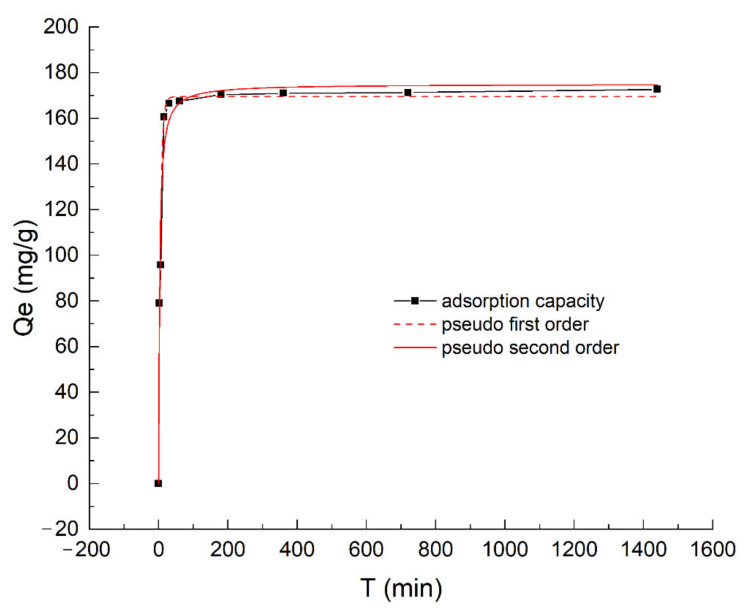
Adsorption kinetics of P on OBC.

The P adsorption kinetics of OBC are illustrated in Figure 2. Clearly, the P adsorption capacity increased with the increase in contact time and reached equilibrium at around 120 min, which was similar to other studies [35,40,41]. Higher P concentration can provide a greater driving force to overcome the mass transfer resistance between liquid phase and the solid adsorbent surface. A greater driving force will accelerate the P adsorption rate. Moreover, P adsorption mainly takes place on the outer surface of OBC by surface-active sites. More surface-active sites are conducive to P adsorption by OBC [42]. During the adsorption process of P by OBC, the concentration of P and the number of surface-active sites both decreased with the increase in adsorption time. Hence, as shown in the Figure 2, P adsorption rate was fast at the beginning, especially within the initial 30 min, then slowed down to zero. The results of this experiment were consistent with previous studies [27,34]. 

The pseudo-first-order and pseudo-second-order kinetics equations as expressed in Equations (4) and (5) were used to fit the experimental data to explore the P adsorption mechanism, respectively [38].

(4) $$\mathrm{Pseudo}-\mathrm{first}-\mathrm{order}:\;\ln\left(Q_{e}-Q_{t}\right)=lnQ_{e}-K_{1}t$$

(5) $$\mathrm{Pseudo}-\mathrm{second}-\mathrm{order}:\;\frac{t}{Q_{t}}=\frac{1}{K_{2}\cdot Q_{e}^{2}}+\frac{1}{Q_{e}}t$$

where $Q_{e}$ (mg/g) and $Q_{t}$ (mg/g) represent the P adsorption capacity at equilibrium and at t time, respectively. $K_{1}$ (min^−1^) and $K_{2}$ (g mg^−1^ min^−1^) are the adsorption rate constants of pseudo-first-order and pseudo-second-order kinetics models, respectively. The corresponding fitting parameters are documented in Table 2. 

It was found that the nonlinear regression coefficients of determination simulated with the pseudo-second-order model were higher than those simulated with the pseudo-first-order model, indicating that the P adsorption by OBC could be better described by the pseudo-second-order model. The pseudo-second-order kinetic model assumes that the removal process of adsorbate is controlled by chemical adsorption, which involves the valence forces where the adsorbent and the adsorbate exchange or share electrons and may form new compounds [40]. Moreover, the pseudo-second-order model also assumes a monolayer adsorption, agreeing with the above isotherm results. 

### 3.2. Effect of Initial Solution pH

**Figure 3:**
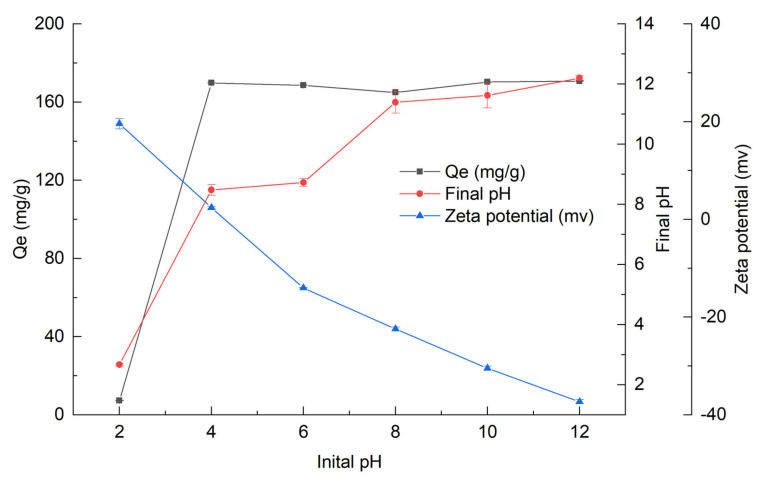
Effect of initial pH on adsorption capacity of OBC, final pH after P adsorption and zeta potential for OBC.

The initial solution pH, as an important factor in the adsorption process, has a close interaction with the form of P in the solution and the surface charge of the adsorbent [41]. The effect of initial solution pH on P adsorption by OBC is shown in Figure 3. At pH 2, the P adsorption capacity of OBC was very low, only about 7.29 mg/g. However, the P adsorption capacity of OBC sharply increased from 7.29 mg/g to 169.82 mg/g with the pH increasing from 2 to 4. Afterwards, with the pH increased from 4 to 12, though the P adsorption capacity of OBC slightly fluctuated, it still maintained high efficiency at about 170 mg/g. The results manifested that OBC had a significant removal efficiency for P in a wide pH range (4–12) from weak acid to base, which would be favorable for its application in realistic water bodies. The final pH of the solution after adsorption is also shown in Figure 3. It was clear that the final pH of the solution had an increasing trend compared to the corresponding initial pH. At low initial pH of 2 and high initial pH of 10 and 12, a small increase was observed, but a larger increase was observed between the pH of 4 and 8. For example, the final solution pH increased to 8.42 for the initial pH of 4, while it only increased to 2.7, 11.68, and 12.19 for the initial pH of 2.0, 10.0, and 2.0, respectively.

The initial solution pH directly determined the form of P, which has a remarkable effect on P adsorption capacity by OBC. The ionization equilibrium of P at different solution pH can be expressed as Equations (6)–(8) [41].

(6) $$\mathrm{pH}=2.16\;{H_{2}\mathrm{PO}}_{4}{}^{-}+H_{2}O\to {\;H_{3}\mathrm{PO}}_{4}{+\mathrm{OH}}^{-}$$

(7) $$\mathrm{pH}=7.20\;{\mathrm{HPO}}_{4}{}^{2-}{+H}_{2}O\to {H_{2}\mathrm{PO}}_{4}{}^{-}{+\mathrm{OH}}^{-}$$

(8) $$\mathrm{pH}=10.30\;{{\mathrm{PO}}_{4}}^{3-}{+H}_{2}O\to {\mathrm{HPO}}_{4}{}^{2-}{+\mathrm{OH}}^{-}$$

According to Equations (6)–(8) and Figure 3, the adsorption capacity of H_2_PO_4_^−^ by OBC was lower than that of HPO_4_^2−^ and PO_4_^3−^, and similar behavior was observed in other studies [34,43]. In addition, the adsorbent surface charge determined by the pH at point of zero charge (pHpzc) of the adsorbent also has a direct influence on its P adsorption capacity. As shown in Figure 3, the pHpzc of OBC sample was about 4.29. When the solution pH value was less than pHpzc (4.29), the surface of OBC would be protonated, and the surface charge of OBC will became positive charge. At this time, P adsorption by OBC mainly was contributed to electrostatic adsorption. When solution pH increased to above pHpzc, the surface of OBC became negatively charged. According to the principle of “like charges repel, unlike charges attract”, a repulsion would exist between OBC and P, so electrostatic adsorption did not work [44]. However, the P adsorption capacity of OBC was still high. This mainly could be attributed to the chemical reaction between P and the CaO and Ca(OH)_2_ produced during the thermal decomposition of CaCO_3_ originated from Oyster shell during OBC preparation process. 

### 3.3. Adsorption Mechanisms

In this study, the characterization of adsorbents before and after P adsorption were analyzed by SEM, FTIR, XRD, and BET to explore the P adsorption mechanism. 

**Figure 4:**
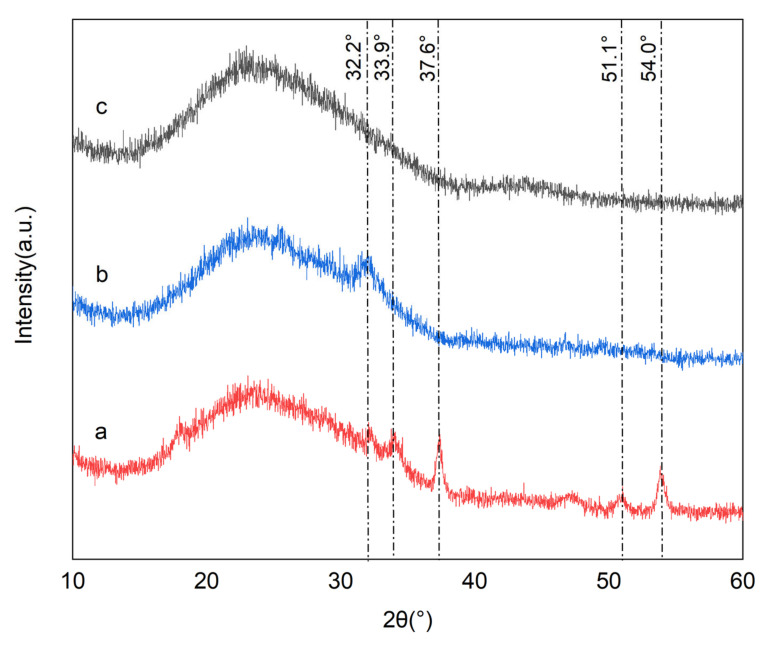
XRD spectra of OBC before P adsorption (**a**), OBC after P adsorption (**b**) and BC (**c**).

The crystallinity of BC, OBC before and after adsorption were analyzed by XRD (Figure 4). As shown in Figure 4, BC, OBC before and after P adsorption exhibited a broad diffraction peak in the range of 20–30°, which implied the amorphous existence of carbonaceous matrix [8]. The XRD spectra of BC was smoother than that of OBC before adsorption, indicating OBC had a high crystallinity and low purity. The diffraction peak of OBC before P adsorption was observed at 2θ = 32.2°, 33.9°, 37.6°, 51.1°, and 54.0°. The diffraction peaks at 2θ = 33.9° and 54.0° could be assigned to Ca(OH)_2_ [34], while the diffraction peaks at 2θ = 32.2°, 37.6°, and 51.1° could be assigned to CaO [34]. The difference in XRD patterns between BC and OBC showed that calcium contained in oyster shell was successfully introduced into OBC with the form of CaO and Ca(OH)_2_. CaO originated from the thermal decomposition of CaCO_3_ during OBC preparation process. Some CaO could react with hydroxyl groups of the biochar or the moisture in the air and form Ca(OH)_2_ [28,37,45]. Moreover, for OBC after P adsorption, the diffraction peaks of Ca(OH)_2_ and CaO disappeared completely, and a new diffraction peaks at 2θ = 32.2° corresponding to Ca_5_(PO_4_)_3_(OH) appeared [34]. These significant changes between OBC before and after P adsorption originate from the formation of hydroxyapatite (HAP) as the following reactions:

(9) $${3\mathrm{PO}}_{4}{}^{3-}{+5\mathrm{Ca}}^{2+}{+\mathrm{OH}}^{-}\to {\;\mathrm{Ca}}_{5}{\left({\mathrm{PO}}_{4}\right)}_{3}\left(\mathrm{OH}\right)↓$$

(10) $${3\mathrm{HPO}}_{4}{}^{2-}{+5\mathrm{Ca}}^{2+}{+4\mathrm{OH}}^{-}\to {\mathrm{Ca}}_{5}{\left({\mathrm{PO}}_{4}\right)}_{3}\left(\mathrm{OH}\right)↓{+3H}_{2}O$$

(11) $${3H}_{2}{{\mathrm{PO}}_{4}}^{-}{+5\mathrm{Ca}}^{2+}{+7\mathrm{OH}}^{-}\to {\mathrm{Ca}}_{5}{\left({\mathrm{PO}}_{4}\right)}_{3}\left(\mathrm{OH}\right)↓{+6H}_{2}O$$

Therefore, the OBC could effectively remove P from the liquid phase. In addition, CaO loaded on the structure of OBC might change its surface charge into positive charge, so the negatively charged P ions in liquid phase could also be removed by electrostatic adsorption, which could be conducive to improving the P adsorption capacity of OBC, too.

**Figure 5:**
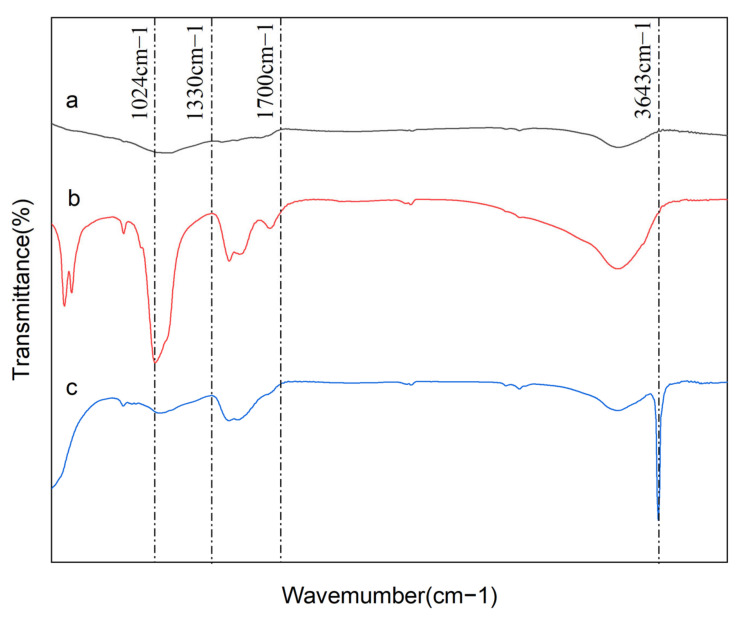
FTIR spectra of BC (**a**) and OBC before (**b**) and after (**c**) P adsorption.

The existing functional groups on the surface of adsorbents was identified by FTIR analyses (Figure 5). As shown in the Figure 5, the spectrum of BC was relatively smooth, which illustrated that the types and numbers of functional groups were very rare. Therefore, the P adsorption capacity of BC was poor. Compared to BC, OBC had a stronger and narrower stretching vibration band of 3641 cm^−1^, which is attributed to -OH from Ca(OH)_2_. However, the stretching −OH vibration band disappeared after P adsorption, and was replaced by a new absorbance band assigned to the bending vibration of P−O at 1024 cm^−1^. This distinct change of spectrum between OBC before and after adsorption could be ascribed to the interaction between the −OH group originated from Ca(OH)_2_ and P. During OBC preparation process, CaO thermally decomposed from the rich CaCO_3_ in the oyster shell could react with the moisture in the air or the hydroxyl groups of the biochar to form abundant Ca(OH)_2_ [28,37,46]. Thus, OBC had a high P adsorption capacity. 

Besides CaCO_3_, the nitrogen-rich organic matter (e.g., protein) contained in oyster shell could form some organic functional groups [14,45,46,47]. Hence, compared with BC, there was a wide stretching vibration range from 1330 cm^−1^ to 1700 cm^−1^ for OBC, including C=C, C=O, C=N, and N–H [27,48]. It was obvious that most of peaks for OBC at these positions became stronger after P adsorption, which might be resulted from the formation of intramolecular and intermolecular hydrogen bonds during the P adsorption process [48]. Therefore, besides chemical precipitation and electrostatic adsorption, hydrogen bonding also played an important role in the removal of P by OBC.

**Figure 6:**
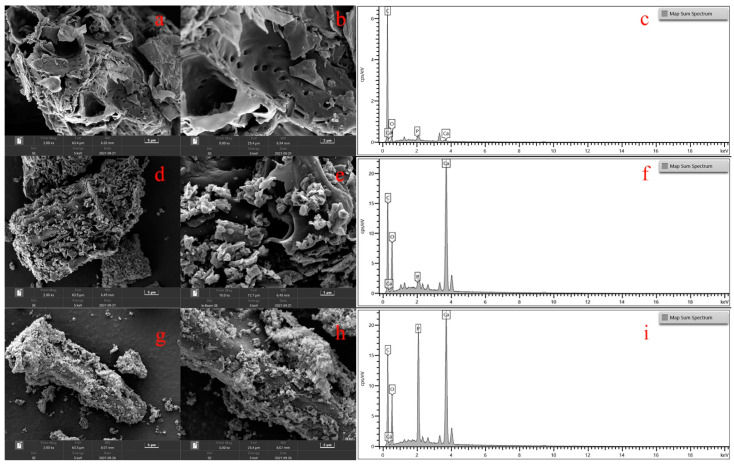
SEM-EDS image of BC (**a**–**c**), OBC before (**d**–**f**) and after (**g**–**i**) P adsorption.

The morphology and structure of BC, OBC before and after P adsorption are shown in Figure 6. The surface of the BC was relatively smoother and rather regular with a highly porous structure, which could provide enough loading space for calcium compounds. The CaCO_3_ contained in the oyster shell was thermally decomposed into CO_2_ and CaO particles during the OBC preparation process [27]. The release of CO_2_ could enlarge the pore size of OBC, resulting in a more porous structure of OBC [34,49]. The structure of OBC shown in Figure 6d,e confirmed this phenomenon. In addition, the generation of CaO could react with the moisture in the air or the hydroxyl groups of the biochar to form Ca(OH)_2_, which is attached to the surface of biochar or embedded in the pores of OBC. Hence, as shown in the Figure 6d,e, numerous accumulated small particles were observed on the surface of OBC [27]. Moreover, the difference in the EDS analysis for BC and OBC also confirmed the successful load of Ca, which was consistent with the XRD analysis. However, after P adsorption, the effective active sites were occupied by a large amount clustered flocculent precipitate, which covered the surface and the pores of OBC. Therefore, the effective active sites sharply decreased, eventually resulting in the adsorption saturation for OBC. From the EDS (Figure 6j) and XRD analyses (Figure 5), it can be confirmed that these precipitates were Ca_5_(PO_4_)_3_OH (HAP). 

**Figure 7:**
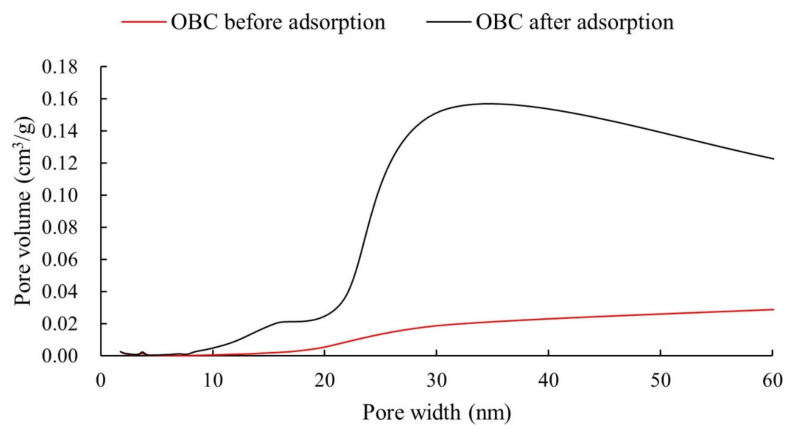
Pore volume distributions of OBC before and after adsorption.

The space pore structure changes of the OBC before and after P adsorption, including BET surface area, pore volume and average pore width, are listed in Table 3. Compared to BC, addition of oyster shell in biochar could increases its BET surface area, pore volume, and average pore width. During the pyrolysis process at high temperature, the abundant CaCO_3_ in oyster shell decomposed into CO_2_, whose release increased the pore size of the biochar [28,35,50]. Moreover, the higher specific surface of the flocculent Ca-P precipitations formed on the surface of OBC led to a higher BET surface area, pore volume, and average pore width for OBC after P adsorption [27]. BJH desorption pore distribution of the OBC before and after P adsorption is shown in Figure 7, showing an obvious redistribution of the space pore structure distributions for OBC [27]. As shown in the Figure 7, the generated Ca-P precipitation is mainly distributed within the pore size range of 20–60 nm. 

### 3.4. Seed Germination and Seedling Growth

**Figure 8:**
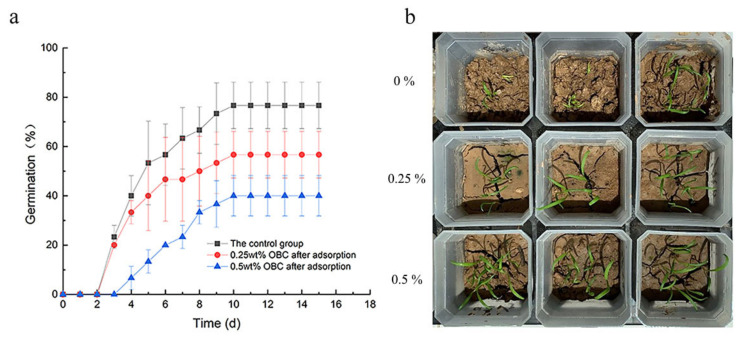
Germination rate of plants (**a**) and images of plant growth (**b**).

The application of OBC after P adsorption as a P fertilizer was studied by a pot experiment. The experimental results are shown in Table 4 and Figure 8. It showed that OBC after P adsorption promoted *Spinacia* germination and growth. Compared with the control experiment only with soil, the germination rate of the addition OBC after P adsorption with 0.25 wt% and 0.5 wt% was increased by 16.67% and 36.67% respectively. The dry weight and wet weight of *Spinacia* with OBC after P adsorption are listed in Table 4, the dry weight and wet weight of *Spinacia* increased with the increase in the addition amount of OBC after P adsorption. These results indicated that the absorbed P by OBC could be released and absorbed by *Spinacia* during its germination and growth process [50]. Hence, the OBC after P adsorption showed great potential to be used as a soil slow-release P fertilizer to improve soil fertility and promote *Spinacia* growth. However, the long-term effectiveness of OBC after P adsorption as a slow-release P fertilizer for different crops with different types of agricultural soils is unclear and should be studied in the future.

## 4. Conclusions

In this study, a novel Ca-modified biochar was prepared by co-pyrolysis oyster shell and peanut shells with mass ratio 1:1. The synthesized Ca-modified biochar was used to recover P from P-rich aqueous solution and demonstrated excellent performance in a wide range pH 4–12. The maximum P adsorption capacity of Ca-modified biochar was about 168.2 mg/g with adsorbent dosage 1 g/L at 28 °C for 2 h, which was 27.6 times higher than that of the unmodified biochar. Characterization analysis of biochar by SEM, XRD, FTIR, and BET indicated that the dominant adsorption mechanism included chemical precipitation, electrostatic adsorption, and hydrogen bonding. OBC after P adsorption exhibited a good ability to be reused as a new slow-release P fertilizer for soil to promote *Spinacia* germination and growth. 

## Figures and Tables

**Table 1:** Fitting parameters of the adsorption isotherm for P on synthetic materials.

Adsorbents	Langmuir Model			Freundlich Model			Temkin Model		
	*K_L_* (L/mg)	*q_max_* (mg/g)	R^2^	*K_F_*	1/*n*	R^2^	$b_{T}\;(J/\mathrm{mol})$	$A_{T}$	R^2^
OBC	7.9578	172.4149	0.987	40.5597	0.2826	0.960	152.4065	236.1026	0.982

**Table 2:** Adsorption kinetic parameters of P on OBC.

Adsorbents	Pseudo-First-Order Model			Pseudo-Second-Order Model		
	*K*_1_ (h^−1^)	*q_e_* (mg/g)	R^2^	*K*_2_ (g mg^−1^ h^−1^)	*q_e_* (mg/g)	R^2^
OBC	0.188	169.466	0.966	1.92 × 10^−3^	172.984	0.975

**Table 3:** Space pore structure parameters of the biochar before and after adsorption.

	BC	OBC before Adsorption	OBC after Adsorption
*S_BET_*(m^2^/g)	67.9042	127.2446	141.5378
Pore volume (cm^3^/g)	0.0938	0.3691	0.5614
Average pore width (nm)	5.5346	12.2830	15.8643

**Table 4:** Plant weight.

	Control Group	0.25 wt% OBC after Adsorption	0.5 wt% OBC after Adsorption
Wet weight (g)	0.1462 ± 0.0284	0.2263 ± 0.0143	0.2597 ± 0.0269
Dry weight (g)	0.0132 ± 0.0008	0.0251 ± 0.012	0.0311 ± 0.0066

## Data Availability

Data is contained within the article.
